# Beer, Wood, and Welfare ‒ The Impact of Improved Stove Use Among Dolo-Beer Breweries

**DOI:** 10.1371/journal.pone.0132603

**Published:** 2015-08-05

**Authors:** Michael Grimm, Jörg Peters

**Affiliations:** 1 Department of Economics, University of Passau, Passau, Germany; 2 International Institute of Social Studies, Erasmus University Rotterdam, Rotterdam, The Netherlands; 3 Institute for the Study of Labor (IZA), Bonn, Germany; 4 Rheinisch-Westfälisches Institut für Wirtschaftsforschung (RWI), Essen, Germany; 5 The African Microeconomic Research Unit (AMERU), University of the Witwatersrand, Johannesburg, South Africa; Universitat Rovira i Virgili, SPAIN

## Abstract

Local beer breweries in Burkina Faso absorb a considerable amount of urban woodfuel demand. We assess the woodfuel savings caused by the adoption of improved brewing stoves by these micro-breweries and estimate the implied welfare effects through the woodfuel market on private households as well as the environmental effect. We find substantial wood savings among the breweries, 36% to 38% if they fully switch to an improved stove. In absolute amounts, they save about 0.176 kg of fuelwood per litre of dolo brewed. These savings imply huge reductions in CO_2_-emissions and reduce the overall demand for woodfuel, which is predominantly used by the poorer strata for cooking purposes. We provide estimates for the price decrease that might result from this and show that the urban poor are likely to benefit. Thus, the intervention under study is an example for a green growth intervention with pro-poor welfare gains – something green growth strategies should look for.

## Introduction

Poverty and environmental hazards are directly related. One of the most striking examples for this is the usage of biomass–mostly firewood and charcoal–for cooking purposes. More than 3 billion people rely on such fuels, because modern cooking fuels like electricity or gas are not affordable or accessible. The provision of woodfuels is associated with a heavy burden for the users. In rural areas where firewood is mostly collected, it induces a substantial work load, in urban areas where charcoal or firewood must be bought, it induces a heavy monetary burden. In addition, the wood extraction and the combustion in mostly very inefficient cooking devices have severe environmental implications (see [1, 2]). First, on the local level the inefficient combustion process leads to smoke emissions that contain harmful pollutants killing 4.3 million people every year according to the World Health Organisation [3]. On the regional level, biomass usage contributes to deforestation and forest degradation. On the global level, burning biomass leads to climate relevant emissions, most notably CO2 and black carbon. Combating such environmental deficits is high on the agenda of international cooperation and national governments. The idea of putting developing countries on an environmentally more sustainable trajectory has made inroads into the rhetoric of aid agency as green growth (see e.g. [4]).

In this paper, we focus on one particular type of green growth strategy: the promotion of investments in energy-efficient production processes of micro-breweries in urban Burkina Faso. The intervention–called FAFASO (*Foyer Amélioré Burkina Faso*)–has been implemented by the German Technical Cooperation (GIZ) and trains masons in the installation of improved brewing stoves facilitates and sensitizes the local beer breweries for the investment in these stoves. In Burkina Faso–country facing substantial deforestation pressures–local beer breweries absorb a considerable amount of urban woodfuel demand–more than 50% according to the Burkinabè Ministry of Environment. The policy intervention under evaluation is a classical green growth intervention and potentially a low-hanging fruit for both the ‘social planner’ and the breweries. For the former, it might be a low-cost opportunity to alleviate deforestation pressures and reduce climate relevant emissions, for the latter it might constitute a profitable investment with a relatively short repayment period. The key research question of this paper is whether these expectations hold true.

In addition to this, our paper probes into the distributional effects of energy efficiency interventions targeting fuels that are primarily used by the poor. Firewood is the main cooking fuel for private households in poor countries and in particular in urban areas where firewood has to be purchased the share of expenditures spent on cooking energy is disproportionally higher for the poorest. Therefore, we also measure some of the indirect effects on households as drastically changing demand patterns for locally traded wood fuel can be expected to affect the prices the urban poor pay for a good they use on a daily basis.

Our analysis is based on two original data sets that we collected among local beer breweries and households in Ouagadougou and Bobo Dioulasso. These local beer breweries are micro-enterprises virtually always run by women, the so-called dolotières. The consumption of the local beer, the dolo, as well as the craft of brewing it is deeply entrenched in the Burkinabè culture. It is usually modestly consumed, but on a very regular basis and by most Burkinabè. The brewing process is very energy and labour intensive and rather an artisanry than an industrial process.

To our knowledge, this paper is the first to examine the direct impact of an energy efficiency intervention targeted at woodfuel consumption in micro-enterprises. We also explore second-round effects through price adjustments on consumers. The latter is particularly interesting as these effects can expected to be highly pro-poor. However, due to data limitations we abstract from further macro-economic responses associated with rebound effects, woodfuel supply reactions and possible adverse effects on woodfuel traders. In that sense our analysis is a partial in its scope and on purpose focuses on those channels that are likely to be pro-poor. Our paper is related to the large literature on improved stoves for cooking (see e.g. [5, 6, 1, 7–11]) that usually focuses on health and environmental impacts as well as direct effects on energy expenditures. Our paper also contributes to the literature on innovation and technology adoption in a context in which credit and insurance markets are incomplete and returns from innovation might be uncertain (see e.g. [12–16]). The paper is also related to the literature that explores the distributional impacts of food price inflation and the inflation of other goods that particularly matter for poor households (see e.g. [17–20]). Finally, the paper contributes to the literature on the role of woodfuels for deforestation and the implied economic costs (see e.g. [21, 22]).

The remainder of the paper is organized as follows. In the next section we provide background information about the business of making ‘dolo’ and the stove intervention under study. In Section 3, we present our data. In Section 4 we explain how we assess direct savings for breweries, the indirect welfare effects for wood-consuming households and the environmental impact. Section 5, we first analyze adoption and then present the results on the wood savings associated with the use of an improved brewing stove and briefly discuss the potential indirect effects. In Section 6 we conclude.

## The Business of Making ‘Dolo’ and the ‘FAFASO’ Intervention

The improved cooking stoves made for breweries–‘Roumdé stoves’ hereafter—are much larger than the household cooking stoves and are made of clay and bricks rather than metal (‘Roumdé’ is the brand name chosen by the German technical cooperation (GIZ), it means ‘the preferred’ in the national language Mooré). These stoves are fixed and typically comprise between two and five huge cauldrons (although different sizes exist), the so-called ‘marmites’ (if made of aluminium) or ‘canaris’ (if made of clay). Aluminium is more widespread in Ouagadougou and clay in Bobo-Dioulasso. In front is a slot to the combustion chamber through which the firewood is loaded; typically by using entire trunks of wood that are by and by moved into the oven. A typical stove easily spans the surface of three to four square metres with the cauldrons arranged symmetrically over this space. In contrast traditional stoves basically consist of a set of cauldrons that is lifted by a few bricks allowing moving firewood under the cauldrons. Some slightly modified versions of these traditional stoves exist, which have some sort of combustion chamber, but are not of the same quality than the Roumdé. Photographs of both the Roumdé and two traditional stoves are shown in the supporting information (S1 File, see Figs A–C in S1 File) to this article.

A Roumdé costs about CFA F 27,500 (EUR 42 if official exchange rate used or EUR 157 if PPP exchange rates are used) without the cauldrons. Aluminium cauldrons (marmite) cost depending on the size between CFA F 20,000 (EUR 30) and CFA F 60,000 (EUR 90) and are hence more expensive than the Roumdé it-self and much more expensive than clay cauldrons, but they also have a much longer life-span than the latter. Cauldrons made of clay often crack if the stove is overheated. Aluminium cauldrons can in principle melt, but it seems that this happens only very rarely. Because changing the cauldrons with a Roumdé is also expensive, since the upper mantle of the stove needs to be opened, some dolotières switch from clay to aluminium when they buy a Roumdé. Typically, the change of cauldrons as well as other maintenance work needs to be done by a mason and costs about CFA F 1,000 to 2,000 (although some dolotières were trained by FAFASO to make the change themselves). Increased maintenance costs are also related to the brittleness of the door of improved stoves. Since dolotières use relatively large trunks of wood, the doors often break; as they would either require to use smaller pieces of wood or at least to fill the stove with much more care. Repairing the doors is expensive. Leaving the door broken in turn or at least not repairing it in a professional manner substantially reduces the efficiency of the stove.

According to the GIZ a Roumdé saves at least 60% to 70% of the firewood needed with a traditional stove for one brewing process. However, it seems that the saving rate goes rapidly down if the improved stove is poorly maintained. In one field test conducted by the *Institut de Recherches en Sciences Appliquées et Technologies* (IRSAT) a damaged improved stove even needed more firewood per litre of dolo than a traditional stove [23], confirming that a rigorous assessment of the effectiveness of such stoves requires a test under real world conditions where conditions include the quality status of the stove and how the stove is used.

FAFASO is implemented under the umbrella of the Dutch-German energy partnership ‘Energising Development’ (EnDev). FAFASO, targets three types of actors: households, social institutions, such as schools and health centres, and microenterprises. We focus on local beer breweries. The FAFASO intervention differs from other earlier improved cookstove (ICS) promotion programmes in Burkina Faso mainly because it does not provide direct subsidies. Instead, it rather focuses on the training of ICS producers (whitesmiths, potters and masons), sensitization, and marketing campaigns. FAFASO started in 2005 to promote ICS in two cities, the capital, Ouagadougou and Bobo-Dioulasso, Burkina Faso’s second largest city. Initially the program started with cooking stoves for private households. In 2008 the GIZ started to train masons in constructing special stoves for dolo breweries that are designed to curb firewood consumption in the brewing process, since the production of dolo needs a lot of energy, typically firewood, because once the basis of the beer, the sorghum, is crushed and ground into a paste (malt), it needs to be boiled for more than a day. The training of masons was first concentrated in communities in the Eastern region of the country and was implemented in collaboration with dolo producer associations. From 2008 onwards such trainings were repeatedly organized. In Ouagadougou and Bobo-Dioulasso, these trainings started in 2010 (in Bobo-Dioulasso six months later than in Ouagadougou). This was accompanied by sensitization campaigns among dolo producers in both cities and in the rural communities around Ouagadougou and by the installation of test stoves in breweries where the dolotières had some model or leader-role (‘*femme leader*’). Further masons were trained in the Centre-Est region.

Table 1 shows the number of installed Dolo stoves in 2010, 2011 and 2012. The number of installations peaked in 2010 in the regions ‘Sud-Ouest’ and ‘Est’ and in 2011 in Ouagadougou and Bobo-Dioulasso. In 2012 the number of installations decreased significantly. By the end of 2012, 2,317 stoves had been installed. The decline from 2012 onwards might be due to market saturation. The early awareness campaigns by the FAFASO seem to have been quite successful, so that by 2012 maybe all those that had made plans to invest in such a stove had already bought one.

**Table 1:** Number of improved dolo stoves (Roumdé) installed by location and year.

Location	2010	2011	2012	Total
Ouagadougou	93	592	181	866
Bobo-Dioulasso	29	336	58	423
Sud-Ouest	280	241	143	664
Est	154	105	105	364
Total	556	1,274	487	2,317

*Source*: FAFASO.

Making dolo is a tradition. The activity is exclusively done by women, typically Christian or animist, since Muslim women are not allowed to make alcohol. The alcohol arises once the boiling of the malt is done. By adding yeast and by letting it ferment, the beer–dolo–is produced. When the dolo is ready, the women typically fill up big plastic barrels of it. They then sell either directly to customers or to other retailers. For the customers, most dolotières have a so-called cabaret, typically some benches to a shady spot outside the courtyard. Usually, the cabaret scene is geared towards simple socializing. Excessive drunkenness is rare. People start passing through around eight or nine in the morning, on their way to work. Others come during the day or on their way back from work. Those who consume in the cabaret drink it from a so-called ‘calabashe’. For take-away the breweries usually use empty soda bottles or plastic containers. A litre bottle of dolo is sold for about CFA F 150 (EUR 0.23). In urban areas the typical brewery is located in a backyard has one or several stoves, additional cauldrons and barrels to stock raw materials, intermediate outputs, residuals and the final product, the dolo. Wood is stocked at the side or outside the yard. The piles of wood can be relatively large, since most breweries purchase wood for several brewings.

## Data

We use three different types of data: (i) dolo breweries survey data, (ii) information drawn from focus group discussions, in-depth interviews with stakeholders and other experts and field visits and (iii) household data on cooking behaviour. In what follows we briefly present each source.

### Survey data on dolo breweries

In 2010 the *Institut de Recherches en Sciences Appliquées et Technologies* (IRSAT) conducted a census to count all dolo breweries in greater Ouagadougou and Bobo-Dioulasso, i.e. including their surrounding (rural) villages. The census revealed that in and around Ouagadougou 2,397 breweries were operating. In and around Bobo-Dioulasso the count was 1,144 breweries [23]. Because the census had been conducted at the end of the rainy season and some breweries temporally close in that period of the year, the actual number might even be a bit higher.

From this list of breweries, IRSAT then randomly selected 219 breweries—158 in and around Ouagadougou and 61 in and around Bobo-Dioulasso. With the help of a dolo producers association, the selected breweries were then contacted and interviewed. Our questionnaire collected information about the socio-demographic characteristics of these breweries and the people working there, the brewing process including wood consumption and about the awareness and possibly use of improved cooking stoves. This information was used by IRSAT to produce a report commissioned by the GIZ to better target and design the FAFASO activities, in particular the promotion of improved stoves for breweries that only started in 2010 [23].

Two years later, in September 2012 we re-interviewed as many as possible breweries from the 2010 sample and expanded the sample by new breweries to compensate for attrition. Hence, in total 261 breweries, 178 in and around Ouagadougou and 83 in and around Bobo-Dioulasso, were visited and interviewed. The interviews were conducted by staff from IRSAT again with support from the association of breweries. Attrition turned out to be quite high. From the 261 breweries, 88 had already been interviewed in 2010. 44 breweries visited in 2010 refused to participate again in the survey, four had stopped their activity and two owners died. For all other breweries the interviewers could not find the owner (or employed staff) during the period they conducted the interviews. Absence was often due to the fact that during this period of the year labour is needed for harvesting. New breweries were randomly drawn from the list of all breweries registered through IRSAT’s census. Table 2 documents the sample compositions in 2010 and 2012.

**Table 2:** Sample composition (2010, 2012, panel).

	Breweries interviewed in…		
	2010	2012	Both years
Ouagadougou	156	178	72
Bobo-Dialousso	61	83	16
Total	217	261	88

*Source*: Own calculations, based on Brewery Surveys 2010 and 2012.

The questionnaire used in 2010, had been enriched by a number of additional questions allowing better to scrutinize the impact of improved stoves on wood consumption. Information on and related to wood consumption was only incompletely collected in 2010. In particular, information related to the stoves in use were asked separately for every stove such as the type of the stove, its condition, its age, its purchase price, the number of cauldrons, the material of the pots and their size. The questionnaire included also more questions about the use of inputs and the awareness of and attitudes towards improved stoves. The questionnaire had been tested in the field prior to the survey.

Wood consumption would ideally be measured by weighing the actual amount of wood used per brewing. Given the large quantities of wood involved and the long duration of the brewing process, we decided to ask the dolotières to provide an estimate of the value of consumed wood. Experts from IRSAT were confident that the dolotières know very well how much they consume and indeed as will be seen below, the provided information satisfies a number of plausibility checks suggesting that measurement error is not a major issue. In fact breweries buy their wood very regularly and hence seem generally to have a good feeling of how much wood they use. The process of brewing is also very repetitive as it follows always the same procedure and recipe. Nevertheless, we can of course not exclude that a small self-reporting bias remains.

Table 3 presents some basic statistics of the interviewed owners of the breweries. As indicated above, dolo is almost exclusively produced by women and hence in our sample are also only women. They are on average around 45 years old. Only a quarter of them have completed primary school. Two-thirds belong to the ethnic group of the Mossi. The remaining third belongs to the group of Bobo, the dominant group in Bobo-Dioulasso. In 2012, 30% of all breweries interviewed were located in rural areas, i.e. outside of the city in one of the neighbouring villages. Most respondents are already for a long time in business, 15 years on average. Overall, the distribution of the characteristics is very stable between 2010 and 2012, suggesting that the sampling of new breweries to replace the drop-outs did not reduce the representativeness of the sample (see below for a more detailed analysis of drop outs).

**Table 3:** Characteristics of respondents.

	2010	2012
Age (years)	43.7	45.9
At least primary completed (= 1)	0.24	0.23
Ethnic group		
Mossi (= 1)	0.67	0.63
Bobo (= 1)	0.25	0.27
In Dolo business (years)	14.4	16.4
Ouagadougou/Centre Region	0.72	0.68
Urban (= 1)		0.30
N	217	261

*Note*: *Urban/rural has not been coded in 2010*

*Source*: Own calculations, based on Brewery Surveys 2010 and 2012.

Table 4 presents descriptive statistics regarding the breweries and the beer production, now only based on the 2012 survey for which the information has been elicited in more detail. We show the characteristics separately for Ouagadougou and Bobo-Dioulasso and for Bobo-Dioulasso also separately for the city, as only there FAFASO has been active. Breweries in Ouagadougou have on average 1.8 stoves. Breweries in Bobo-Dioulasso are somewhat smaller. In Ouagadougou 0.8 stoves, i.e. less than 50% of these stoves are Roumdé stoves. In Bobo-Dioulasso only 0.3 stoves are Roumdé stoves, i.e. less than 25%. However, if the count is limited to the city of Bobo-Dioulasso, the average number is 0.8, which is then more than 50%. In Ouagadougou and Bobo-Dioulasso respectively 0.85 and 0.42 stoves fall into the category ‘improved traditional stoves’ (0.62 in Bobo-Dioulasso city). 38% of the Ouagadougou sample and 17% of the Bobo-Dioulasso sample use only a Roumdé. Stoves in Ouagadougou typically have four cauldrons, in Bobo-Dioulasso even five or six. Whereas in Ouagadougou aluminium cauldrons are more common; in Bobo-Dioulasso clay cauldrons are more frequently used. In Bobo-Dioulasso the common view among consumers is that dolo beer only has its authentic taste if it is brewed in clay cauldrons. The reported age of the stove (not necessarily the cauldrons) is exceeds eight years on average. The enumerators classified most stoves as being in a good condition, in particular in Bobo-Dioulasso; some have cracks or a broken door. Doors typically break when complete trunks of trees are little by little moved into the stove. Moreover, the high temperature that is achieved can damage the cauldrons. Another typical cause of damage is rain and dogs that search protection in the stoves when not in use. Given the simplicity of traditional stoves, they are less subject to obvious damages.

**Table 4:** Characteristics of breweries in 2012.

	Ouaga		Bobo		Bobo city only	
	mean	sd	mean	sd	mean	sd
Number of paid employees	1.09	2.05	0.37	0.74	1.00	0.98
Number of stoves	1.79	0.92	0.48	0.50	1.50	0.58
Distribution of stoves by type						
Number of traditional stoves	0.12	0.45	0.48	0.50	0.12	0.33
Number of improved traditional stoves	0.85	0.91	0.42	0.59	0.62	0.75
Number of Roumdé stoves	0.81	1.02	0.27	0.61	0.77	0.82
Share of breweries with at least one Roumdé	0.49		0.18		0.54	0.51
Number of cauldrons	6.58	3.52	5.89	2.65	8.08	3.07
Type of cauldrons (shares of stoves)						
Aluminium	0.93	0.25	0.01	0.11	0	0
Clay	0.04	0.21	0.98	0.15	1.00	-
Age of stove	8.51	12.41	10.34	9.58	9.38	11.23
Condition of stoves (shares of stoves)						
Good	0.50	0.44	0.85	0.35	0.61	0.48
Cracks	0.36	0.45	0.11	0.30	0.31	0.43
Shaby	0.14	0.31	0.04	0.16	0.08	0.20
Number of brewings per week	1.99	0.85	1.71	1.60	1.58	0.88
Share of brewing days by type of stove						
Improved traditional stove	0.51	0.42	0.95	0.48	0.38	0.50
Roumdé stove	0.44	0.48	0.17	0.38	0.50	0.51
Share breweries using only improved Roumdé	0.38		0.17		0.50	0.51
Quantity of Dolo per brewing (in liter)	368.91	277.80	159.45	79.24	217.50	95.43
Quantity of malt per brewing (in kg)	85.37	77.72	41.26	16.77	57.24	18.55
Quantity of water per brewing (in barrel)	7.26	8.08	2.70	1.07	3.65	0.98
Expenditure for firewood per brewing*	8,956.90	9,939.61	4,149.67	2,968.83	7,375.00	2,096.72
Quantity of wood per brewing (in kg)**	179.14	198.79	82.99	59.38	147.50	41.93
Av. quant. of wood per liter Dolo (kg)	0.46	0.28	0.53	0.37	0.78	0.32
Wood delivery (share of breweries)						
Collecting or cutting wood	0.02		0.08		0	0
Buys in small quantities	0.22		0.34		0.35	0.49
By cart	0.40		0.46		0.23	0.43
By lorry	0.03		0.01		0.38	0.20
By truck	0.32		0.12		0.38	0.50
Number of observations	178		83		26	

*Note*: *Not counting those who collect or cut their own fuel wood.

** Quantity derived from reported expenditures assuming an average price of wood per kg of CFA F 50. In Bobo-Dialousso marketing campaigns and training activities of masons have been limited to the city of Bobo-Dialousso that is why we show all statistics also separately for the city of Bobo-Dialousso.

*Source*: Own calculations, based on Brewery Survey 2012.

As can be seen in Table 4, most breweries brew twice a week. The average brewing is much larger in Ouagadougou compared to Bobo-Dioulasso. In Ouagadougou almost 370 litres are produced with one brewing. This requires as input about 85kg of malt and 7 barrels of water. The water-malt ratio determines the quality of the beer and also has an important influence on the required quantity of wood. In Bobo-Dioulasso many breweries produce their own malt and use less water; hence their beer has a higher concentration compared to the beer produced in Ouagadougou. The average brewery in Ouagadougou has a monthly turnover of about CFA F 300,000 to CFA F 600,000 (about EUR 500 to EUR 1,000) assuming that a litre of dolo is sold at CFA F 100 to CFA F 200. Wood and other intermediate inputs (in particular malt and water) account for about EUR 200, such that the average value added (including labour, land and capital costs) that is generated is in the context given quite remarkable, even if the variance around the mean is substantial. The survey did not directly ask for turnover, value added or profits as most dolotières would not accept to give an answer. Hence, these numbers are simply derived from the information about the quantity of dolo produced, the average price per litre and the information about some cost categories.

On average, a brewing in Ouagadougou requires wood of a value of about CFA F 8,957 (or EUR 13.70) or CFA F 24.2 per litre of dolo. In Bobo-Dioulasso we find an average of CFA F 25 per litre (CFA F 34 per litre in Bobo-Dioulasso city). Beyond possible efficiency differences, there are at least two additional factors affecting the cost per litre: On the one hand, wood is a bit cheaper in Bobo-Dioulasso compared to Ouagadougou. On the other hand, breweries in Bobo-Dioulasso use different stoves and cauldrons and buy, as can be seen at the end of Table 4, more frequently their wood in smaller quantities, which typically means they have to pay a higher unit price compared to a larger purchase. In Ouagadougou about 32% of all breweries get their wood by truck and hence have typically a huge pile of wood they take from. One reason why breweries decide to buy in small quantities despite the higher price is that this prevents, at least in the rainy season, the wood from getting wet. In the rural part of Bobo-Dioulasso, some of the smaller breweries still collect or cut their own wood.

### Focus group discussions, expert interviews and field visits

To complement the information drawn from the representative survey, we undertook intensive field work before and after the implementation of the brewery survey. Prior to the brewery survey, we interviewed the GIZ staff managing the project, project collaborators, a group of trained masons and a dolo producer association. Moreover, we visited more than ten breweries in Ouagadougou and Bobo-Dioulasso for in-depth interviews. The gathered information allowed getting a better understanding of the organization and process of dolo production, to adequately design the questionnaire of the survey and to enrich and complement the results from the quantitative impact assessment based on the survey data.

### Survey data on woodfuel consuming households

To illustrate the welfare effects that arise for woodfuel consuming households as a consequence of a reduced price for woodfuel, we use data from a specific household survey that we conducted between February and March 2011 in Ouagadougou and Bobo-Dioulasso [24]. Here we just use the sample for Ouagadougou which covers 892 households. This sample is representative for the population of Ouagadougou except the roughly five percent richest households. The surveys main purpose was to assess the effectiveness of improved cook stove use among private households. The dataset includes information about total expenditure per capita and expenditure per capita for woodfuels, cooking energy and energy as a whole. Woodfuel consumption used for cooking–wood and charcoal–was also measured in quantity. Households were asked to specify and show the amount of fuel used with that particular dish, which the enumerators who were equipped with weigh scales weighed then. In combination with information collected on the number and type of dishes cooked per week, the weekly wood consumption can be determined.

### Ethical issues

To undertake this survey no particular ethical approval was necessary. This was neither a requirement of the institutions we are affiliated with, neither of the Netherland’s Ministry of Foreign Affairs, which financed the data collection, nor was it a requirement by the Burkinabè authorities in particular the Ministry of Research and Innovation. There a three reasons for this. First, participation in the survey was voluntary; all breweries were before the start of the interview informed about the content of the questionnaire and the purpose of the data collection. Only if they gave their verbal consent, the interview was undertaken. They could also at any time of the interview stop the interview and not participate in the survey (and ask that all data already collected is deleted); second, the questionnaire does not contain any intrusive question, it only collected information about basic socio-demographic characteristics and a few key variables in relation to the production of beer, in particular the use of firewood. We did not include questions on household income, health or other personal matters; third, because the survey has been implemented by a public research institute operating under the aegis of the Burkinabè Ministry of Research and Innovation.

To further ensure that the survey did not violate any ethical norm, a meeting was held before the start of the survey with a local association of beer-breweries that is representing the breweries we interviewed. This meeting took place in September 2012 in Ouagadougou. The president of the association, other board members and a dozen women owning and running a dolo-brewery attended the meeting. During that meeting the questionnaire was presented and discussed and the purpose of the survey was explained. There was no opposition to the survey, to the contrary, the association was convinced by the added value such a study can create and fully supported the data collection. Moreover, we presented the survey also in meetings to the Ministry of Energy and to the Ministry of Environment.

### Methodological issues and theoretical thoughts

Our assessment will focus on three types of effects. First, reduced wood consumption and hence reduced production costs for dolo breweries; second, a reduced price of fuel wood for consumers of household cooking energy; and, third, an environmental benefit through reduced deforestation and lower CO_2_ emission. In what follows we explain very briefly how we account for each of these three effects.

### Direct effects on breweries

To provide an assessment of the direct benefits accruing to Roumdé users, we focus on woodfuel savings per litre of dolo brewed and changes in monthly profits. In principle, a straightforward approach to obtain this information could be to undertake a controlled cooking (or brewing) test (CCT). Here, the same amount of dolo beer is prepared using a traditional stove and a Roumdé. However, such tests cannot provide more than a technical benchmark of the potential savings associated with the use of an improved stove, since the effective savings in real-world breweries might deviate from such tests for various reasons. First, breweries may simultaneously use improved and traditional ones. Second, it is unlikely that a dolotière in a CCT under observation behaves as she would behave under day-to-day conditions (known as the Hawthorne effect); for example, in reality the dolotière may do a number of activities simultaneously and, hence, cannot dedicate the same attention to her stove as a brewer in a controlled cooking test. Third, as mentioned above, the effectiveness of a stove may decline over time due to inappropriate maintenance.

Hence, in order to assess the effective savings, a large representative survey which captures the diversity of real-world cooking practices is required. A major problem that needs to be overcome is non-random-selection into the treatment group, i.e. the users of Roumdé stoves may systematically differ along a number of characteristics from non-Roumdé users. To the extent these characteristics are correlated with wood consumption, this leads to biased impact estimates, because differences in wood consumption are falsely attributed to the Roumdé. We try to redress at least the bias that stems from observable differences through the use of ‘propensity score matching (PSM)’. Because in our case the sample size is relatively small and the impact assessment needs to be done separately for different pairs of stoves (Roumdé stoves vs. traditional stoves and Roumdé stoves vs. improved traditional stoves) the standard matching approach is not feasible as the number of cases in the various treatment and control groups would be too small. In this case it is better to rely on a special variant of the matching approach, proposed by Hirano, Imbens and Ridder [25] and further discussed in Hirano and Imbens [26] in which the inverse of the propensity score is used to weight each observation in the treated group, and the inverse of one minus the propensity score (i.e. the propensity of not being in the treated group) in the control (see [25, 27]). This formula is used to determine the average treatment effect, whereas Brunell and DiNardo [28] provide an extension thereof for the treatment effect on the treated (see below), which will be used in this study. Weighting has the advantage of including all the available data. The risk is, as shown by Freedman and Berk [29] that weighting may increase random error in the estimates, which leads to a downward bias of the estimated standard errors, even if the selection mechanism is well understood.

The implementation of the procedure involved the following steps. First, we estimate a probit model of being a user of a Roumdé stove:

$${\mathrm{Pr}}_{i}(T_{i}^{}=1)=\theta (\beta_{0}+Z_{i}^{'}\beta_{l}+\omega_{i}),$$ (1)

where the dependent variable is the binary outcome of a brewery *i* having an ICS. The underlying latent variable is the conditional probability of having an ICS. The matrix stands for a set of observable characteristics *Z* explaining stove ownership, such as the number of years the dolotière is already in business, her age, age squared, education and her location. The vector *ß* are the associated effects that are estimated. ω stands for the error term and *ϴ* stands for the cumulative standard normal distribution function, i.e. the underlying probability distribution in a probit model.

Formally, the propensity score is defined as

$$e_{i}(Z_{i})={\mathrm{Pr}}_{i}(T_{i}^{}=1|Z_{i})\;\text{with}\;0<e_{i}(Z_{i})<1.$$ (2)

To attain the average treatment effect on the treated, weights can be computed from these propensity scores as outlined in Brunell and DiNardo [28] for both treatment and control observations, denominated *μ* ^*Ti = 1*^ and *μ* ^*C*^ respectively:

$$\mu_{i}^{T=1}=1\;\text{and}\;\mu^{C}=\frac{Pr(T=1\;|Z)}{1-Pr(T=1\;|Z)}\times \frac{p^{C}}{p^{T}},$$ (3)

where *p* ^*T*^ to the fraction of treatment observations and *p* ^*C*^ to the fraction of control observations. S1 Table shows the differences in the household characteristics used to estimate the probit model above before and after reweighting. Two sets of weights are used. One does exclude the other include the quantity of dolo produced. This is done, since on the one hand the quantity of dolo produced is an important correlate of adoption, on the other hand it cannot be excluded that the quantity of dolo produced is altered following the adoption of a Roumdé. Hence, results are shown using both sets of weights. It can be seen that the reweighing procedure leads to an almost perfect balance; none of the differences between the group of owners and non-owners is statistically significant anymore. The impact evaluation is then based on the following regression model:

$$\ln\;{\tilde{Y}}_{i}=\beta_{0}+\beta_{1}{\tilde{ITS}}_{i}+\beta_{2}{\tilde{IS}}_{i}+\beta_{3}{\tilde{X}}_{i}+\beta_{4}{\tilde{Z}}_{i}+u_{i},$$ (4)

where ln ${\tilde{Y}}_{i}$ stands for the outcome of interest: expenditure for firewood per brewing. The tilde indicates that all observations are reweighed with the propensity score-based weights. ${\tilde{ITS}}_{i}$ and ${\tilde{IS}}_{i}$ are indicator variables taking the value one if a given brewery uses an improved traditional or a Roumdé stove respectively or alternatively are shares measuring the share of brewing days that fall on improved traditional and improved stoves respectively. Hence, *β*_*1*_ and *β*_*2*_ are the main coefficients of interest, the saving rates associated with these two types of stoves. The saving rates are always in relation to traditional stoves. ${\tilde{X}}_{i}$ stands for a vector of characteristics relative to the brewery and the observed brewing such as the condition of the used stoves, the number of cauldrons, the quantity of dolo produced per liter, the quantities of malt and water used and the mode of wood provision. As above, ${\tilde{Z}}_{i}$ stands for a vector of characteristics of the dolotière. The term *u*_*i*_ stands for the error term.

Yet, since with a matching estimator a bias due to unobserved heterogeneity can never be ruled out, even if both groups are balanced across a large number of observable characteristics, we also test the robustness of our findings with a difference-in-difference estimator, i.e. we compare the changes in wood consumption over time for those that adopted between both surveys a Roumdé and those who did not. The few breweries that had already an improved stove in the 2010 survey are removed from the sample. The double-difference estimator can, in contrast to the matching estimator, also account for unobservable variables as long as they are constant over time, such as for instance astuteness. The diff-in-diff estimator can be calculated non-parametrically or in a parametric regression framework thus allowing controlling for observed time-varying characteristics that could still lead to a bias if omitted. Hence, the regression can be specified as follows:

$$\ln\;Y_{it}=\beta_{0}+\beta_{1}IS_{it}\times t_{t}^{2012}+\beta_{2}t_{t}^{2012}+\beta_{3}IS_{i}+\beta_{4}X_{it}+\beta_{4}Z_{i}+u_{it},$$ (5)

where the variables follow the same notation than above. The subscript *t* indicates time. $t_{t}^{2012}$ is an indicator variable taking the value one if a given observation is made in 2012. The coefficient of interest, the saving rate associated with the use of an improved stove, is given by *β*_*1*_, the effect of the interaction effect of treatment and time conditional of time effects and being in the treatment group.

Since the 2010 survey does not allow distinguishing traditional from improved traditional stoves, Eq (5) does not differentiate between improved traditional stoves and traditional stoves. Moreover, given the small sample size of the panel and its short horizon with just two waves (the panel includes only 88 breweries, given further missing information in outcomes and/or some of the explanatory variables, the diff-in-diff estimator is based on 66 observations), individual fixed-effects cannot be added to Eq (5), i.e. only time-constant heterogeneity between users and non-users is removed. However, a limited set of observed time-constant characteristics, *Z*_*i*_, can be added to the list of regressors to capture some of the remaining within-group heterogeneity.

A strong implicit assumption of the difference-in-difference estimator is that both groups would have evolved in the same way in absence of the program (parallel trend assumption). Another drawback specific to the case under study is that only a relatively small sub-sample of all dolotières has been interviewed in both years. This sub-sample may not be representative of all dolotières. However, representativeness can be tested by regressing an indicator variable ‘being surveyed in both years’ on a set of characteristics observed in 2010. We use as characteristics that may either be correlated with adoption of a Roumdé or with stove usage behavior and thus the ability to realize savings with an improved stove. We include the age of the dolotière, her education, her ethnic affiliation, how long she is in business, her location, whether she has already an improved stove or at least knows about this technology, the quantity of dolo produced per month and whether the brewery does also retailing.

S2 Table shows the result of such a regression. It can be seen that most of the included explanatory variables are insignificant, except education, size of the brewery as measured by the quantity of dolo made per brewing as well as whether dolotières know already about improved stove or even have already one in 2010 (which is rare). The fact that larger breweries, breweries that do also retailing, breweries that either experiment or at least think about adoption of a Roumdé as well as more educated dolotières have a higher chance of dropping out may imply that we underestimate the saving rate associated with the Roumdé if these seemingly more professional breweries at the end would also make more often, and better, use of the technology, but this is of course just speculation and is difficult to verify. To control for this bias to some extent we include the size of breweries and education as a controls in our impact assessment.

The principal outcome indicator we focus on is the quantity of fuel wood used per brewing process evaluated at its market price. The ‘treatment’ is coded in two different ways: either by a binary variable ‘having or not having an improved dolo stove’ or by a variable measuring the share of stove-days per brewing process that are provided by improved dolo stoves. If for example a brewery uses two stoves for production and one brewing takes two days over which both stoves are continuously in use then each stove provides two stove-days. If one of the two stoves is an improved stove, the share of stove-days provided by an improved stove is 50%. Using the binary variable it is possible to estimate the percentage reduction of fuel wood per brewing process if an improved dolo stove is in use. Using the share variable, it is possible to estimate more precisely the relative reduction of fuel wood consumption as the number of improved stove-days increases. The concept of stove-days is a better concept than the binary variable of an improved stove for breweries that work with different stoves simultaneously. Since, the quantity of dolo produced per brewing differs across breweries, as do the quantities of malt and water used, the quality of the stove and so on; these factors need to be included in the estimations. Eventually, this allows computing the average savings for Roumdé users per litre of dolo made.

### Externalities on fuel-wood consuming households

To estimate the impact of the reduced price for fuel-wood induced by the adoption of improved stoves and the resulting fuel-wood savings, we calculate the change in wood consumption among dolo breweries (*∆D*) and derive from this—making alternative assumptions on the price elasticity of demand—the price change (d*p*) that is necessary to absorb the wood saved by dolo breweries We then simply estimate the welfare effect resulting from savings in wood expenditure, d*W*, by multiplying for each household in our sample the induced price change, d*p*, with the quantity of wood consumed, per household (see S2 File).

Since we are not only interested in the total welfare effect or the average welfare effect per household, but also in whether the price change affects poorer households more, we compute hypothetical benefits across the entire expenditure distribution. This assessment ignores rebound effects among breweries (if they increased output following reduced costs), which could again increase fuel wood consumption. However, given that the market for local beer is quite saturated, the output elasticity should be relatively weak. Possibly, more relevant is that we ignore reductions in wood supply that might follow in the medium term the reduction in demand and would lead to an increase in prices. Therefore, the distributional effects we estimate may decrease in the medium and long run. There might also be adverse welfare effects on the traders of woodfuel, but given their position in the income distribution, this is unlikely to reduce the pro-poorness of such price effects.

### Environmental benefits

The consumption of firewood contributes to deforestation and forest degradation if fuelwood demand outpaces supply by forests. While there has been a long discussion about the extent to which firewood collection in facts leads to deforestation (see [10] for an overview), there is compelling evidence that at least charcoal contributes substantially to deforestation. The reason is that charcoal cannot be produced out of the dead wood and small branches that are mostly collected by households for cooking purposes. Charcoal production requires larger trunks that have to be cut.

A further implication of firewood usage is the emission of climate relevant substances. The emission of CO_2_ that is induced by the combustion of wood is just as high as the amount of CO_2_ that has been sequestered by the tree in the growing process. If only as much wood is extracted from forests as is produced by the natural growing process, the combustion of woodfuels is CO_2_ neutral. Therefore, woodfuel usage only contributes to the net increase in atmospheric CO_2_ to the extent that the wood is extracted in a non-sustainable manner leading to a loss of carbon sinks via deforestation or forest degradation ([30] estimates that net land-use change, mainly deforestation, is responsible for about 10% of the total anthropogenic CO_2_ emissions). This obviously depends on factors like biomass production and population density and is hence due to geographical variation, but for many regions it can be expected that fuelwoods are not extracted sustainably and thus firewood usage contributes to net emissions of CO_2_ [31, 2, 32]. In addition to CO_2_, the combustion of biomass based fuels is the dominant source of black carbon emissions [33]. Black carbon, if gathered in high concentrations in the atmosphere, absorbs sunlight and in this way contributes substantially to short-term warming processes [34]. Shindell et al. [35] identify the reduction of firewood consumption for cooking purposes as a promising quick win against short-term climate change processes, because unlike classical climate gases such as CO_2_ the short-lived nature of black carbon suggests that strong immediate action will generate immediate reduction of warming processes (see also [36, 37]).

While it is obviously beyond the scope of this study to quantify the effects of the reduction in firewood consumption on forests and black carbon emissions, below we will present a conservative calculation of reduction in CO_2_-emissions based on the methodology that is applied for the clean development mechanism (CDM), developed by the United Nations Framework Convention on Climate Change (UNFCCC) [38].

## Results

### The adoption of improved cooking stoves

In a first step a probit regression model is used to analyze the correlation between a range of socio-economic characteristics of the dolotières and characteristics of their breweries and the adoption of a Roumdé. In a second step, the role of these determinants and other factors are further scrutinized using the insights from the field visits, in-depth interviews and focus group discussions.

Theoretically, one would expect that adoption depends on at least four sets of variables: First, it should depend on the degree of energy inefficiency in the before-situation, i.e. breweries that have a high consumption of firewood per litre of produced dolo should gain most from the adoption of an improved stove. Second, adoption is also the more beneficial the higher the price of firewood. Third, adoption should depend on access to information, the intensity of marketing campaigns, i.e. dolotières need to be aware that improved stoves exist and what their savings potential is. Access to information should in turn be related to education, age, location and the interaction with other Dolotières. Fourth, it should depend on the dolotière’s ability-to-pay and her access to credit. Moreover there are additional socio-psychological drivers such as the discount rate, risk aversion, conformity and peer pressure related to all these sets of variables which are however more difficult to observe and hence to account for. Finally, dolotières may have to cater different types of clients with different taste preferences and this may also influence the decision to adopt an improved stove. For a more detailed discussion of drivers of fuel choice and adoption of improved cooking stoves see e.g. [39], [40], [41] and [42].

Based on these considerations, the following explanatory variables are included in the quantitative analysis; a subset of them has also been used to implement the matching procedure: the age and age squared of the dolotière, her education, the number of years she is in business, the quantity of dolo she produces per brewing process as well as binary variables indicating whether the brewery is in Ouagadougou or in Bobo-Dioulasso and whether it is located in the urban area or outside the town in a rural community. The survey does not contain good proxies for wealth or even access to credit, as questions seeking for information on wealth would have decreased their cooperativeness to accurately conduct the interview. However, from other studies that investigate the investment behaviour of informal firms in Ouagadougou and other West-African agglomerations, it is known that access to capital is generally an important constraint [43]. The results of the probit model are shown in Table 5. Given that the quantity of dolo produced per brewing may change with the adoption of an improved stove, and hence the quantity produced has to be considered as endogenous, two sets of regressions are presented one with the quantity of dolo made on the right-hand-side and one without.

**Table 5:** Uptake of Roumdé stoves, probit model, marginal effects.

Dep. Var.: Uses a Roumdé stove	Coeff.	Coeff.
	(S.E.)	(S.E.)
Ln quantity of Dolo per brewing (in liter)		0.199
		(0.070)***
Age dolotière	0.008	0.008
	(0.022)	(0.022)
Age dolotière (sq.)	0.000	0.000
	(0.000)	(0.000)
At least primary completed (= 1)	0.200	0.170
	(0.078)**	(0.080)**
Mossi (= 1)	0.214	0.227
	(0.121)*	(0.123)*
Bobo (= 1)	0.272	0.304
	(0.185)	(0.183)*
In Dolo business (years)	0.030	0.024
	(0.012)**	(0.012)*
In Dolo business (years) (sq.)	-0.001	0.000
	(0.000)*	(0.000)
Ouagadougou/Centre Region	0.600	0.542
	(0.089)***	(0.103)***
Urban (= 1)	0.774	0.740
	(0.104)***	(0.117)***
Ouagad. x Urban (Interaction)	-0.547	-0.552
	(0.051)***	(0.051)***
Pseudo R2	0.236	0.261
N	253	253

*Notes*: The coefficients show marginal effects, i.e. the change in the probability of uptake for a one unit-change in the explanatory variable (or a change from 0 to 1 for binary categorical variables).

* significant at 10%

** significant at 5%

*** significant at 1%. Robust standard errors in parentheses.

*Source*: Own estimations, based on Brewery Survey in 2012.

The marginal effects shown in Column (1) of Table 5, suggest that the probability of adoption is higher by 20% if the dolotière has at least completed primary education. The number of years in business has also a significant effect. Each additional year in business increases the probability of adoption by about 7%. However, the squared term is negative suggesting that this effect decreases with age. Adoption in the Ouagadougou region is much higher than in the Bobo-Dioulasso region, as mentioned above, this advantage mainly relates to the rural area of Ouagadougou. If the quantity of dolo is added to the list of regressors, the results suggest that for every percentage increase in the quantity of dolo produced, the probability of adoption increases by 0.2%. The age of the dolotière does not have a significant effect on adoption. The ethnic affiliation does have a weakly significant effect, but given the dominance of Mossi in Ouagadougou and Bobo in Bobo-Dioulasso it is difficult to disentangle this effect from the location effect.

The used questionnaire also included a module asking the dolotières without a Roumdé whether they know the Roumdé and if so, where they have heard about it. 60% of the non-users reported that they know the Roumdé. Most of them, about 79%, have heard about it from neighbors and other dolotières. Another 10% know the Roumdé from FAFASO marketing campaigns and 6% have heard about them from their masons.

Through in-depth interviews and focus group discussions, the determinants of uptake have been further explored. In general dolotières did not doubt the higher efficiency of a Roumdé, although they often mentioned that it requires a lot of effort to train staff in a way that less wood than with a traditional stove is consumed. Moreover, dolotières frequently mentioned that maintenance costs are a problem and that the investment costs of adoption are for many simply too high. Switching to aluminium cauldrons would solve the problem of maintenance somehow, as they have a longer life-span (a couple of years, it is difficult to provide an exact number here, as it depends a lot on how and how often they are used.), but it adds substantially to the investment that needs to be made up front. Depending on the size an aluminium cauldron costs between CFA F 20,000/30,000 (small) and CFA F 50,000/60,000 (large).

Moreover, it was also mentioned by many respondents that traditional stoves typically have five to six cauldrons, whereas Roumdés have only four. Hence, a Roumdé offers less brewing capacity but needs more or less the same space. A final issue which can further explain lower take-up rates around Bobo-Dioulasso (besides lower program activity) is that 8% of all breweries still report to collect their own firewood (compared to 2% in and around Ouagadougou). As a consequence, the efficiency of their stove in terms of wood consumption is less of an issue and, hence, the incentive to buy a Roumdé might be somewhat lower depending on how they perceive the time cost implied by the firewood collection.

### Wood savings and rate of return

As explained above, we provide two alternative estimates: First, one based on the difference in firewood consumption between Roumdé users and non-users in 2012, where all breweries without a Roumdé are weighted according to their empirical propensity to adopt a Roumdé; second, the difference-in-differences estimate of wood consumption comparing those breweries that adopted an ICS between 2010 and 2012 and those that did not.

The key results from the econometric assessment are shown in Table 6 and in full detail in S3 Table. The results suggest that breweries that use at least one Roumdé (they may still use traditional stoves in addition in case they use more than one stove) spend about 18% less on firewood per brewing process than breweries that use a traditional or improved traditional stoves (but no Roumdé stove). These estimates control for the quality of the stove, for the quantity of dolo per brewing, the quantity of malt used, the quantity of water used, the number of cauldrons used, the source of the firewood purchase, the age and age squared of the dolotière, her education, her ethnicity, the time she is already in business and indicator variables for Ouagadougou, urban areas, and the corresponding interaction effect. The difference-in-difference estimator is similar in magnitude, but less precisely estimated, mainly due to the very small sample size. Again, because, the 2010 survey does not allow distinguishing traditional and improved traditional stoves, both categories are lumped together in the reference category. Hence what is estimated are savings relative to a mix of both types of stoves. These savings should be somewhat lower than those that one would obtain if measured in comparison to traditional stoves only.

**Table 6:** Impact of Roumdé usage on firewood consumption in CFA F (log).

	OLS-CS 2012	OLS-CS 2012	Diff-in-Diff	Diff-in-Diff
	PS-weights I	PS-weights II	non-param.	parametric
Uses a traditional/improved traditional stove	Ref.	Ref.	Ref.	Ref.
Uses a Roumdé stove	-0.182	-0.187	-0.213	-0.143
	(0.064)***	(0.064)***	(0.612)	(0.340)
*N*	*236*	*236*	*66*	*66*
Share of brewing-stove-days	Ref.	Ref.		
with a traditional stove				
Share of brewing-stove-days	-.200	-0.214		
with an improved traditional stove	(0.168)	(0.154)		
Share of brewing-stove-days	-0.358	-0.376		
with a Roumdé stove	(0.163)**	(0.153)**		
*N*	*236*	*236*		

*Notes*: The results of each regression are shown in detail in Annex 4.

* significant at 10%

** significant at 5%

*** significant at 1%. Robust standard errors in parentheses.

*Source*: Own estimations, based on Brewery Surveys 2010 and 2012.

Since a non-negligible share of breweries use the Roumdé and traditional stoves simultaneously, these estimates provide for an accurate assessment of how much more firewood is consumed in non-Roumdé using breweries. However, it does not provide for an estimation of how much firewood could be saved if all brewing processes were prepared on a Roumdé stove. For this purpose, the share of stove-days that fall on a Roumdé stove is used as treatment variable. This is only possible with the 2012 data set. Moreover, the reference category is now also split into days that fall on a traditional stove and days that fall on an improved traditional stove. The corresponding results are shown in the lower part of Table 6. The estimated coefficient ranges between 0.36 and 0.38 depending on which version of the propensity score weights is used. This implies that a brewery that would switch from no Roumdé stove to only Roumdé stoves, realizes savings in firewood per brewing by about 36% to 38%. This saving rate is by roughly 40% smaller than what could technically be achieved (0.37/0.60). Remarkably, improved traditional stoves are at least associated with a saving rate of about 20%. Yet, the estimate is not very precise (p = 16.7 to p = 23.6).

Taken together the results show that the estimates are quite robust to the exact specification and weights chosen. However, a few potential sources of bias need to be discussed. First, the estimate might be downward biased, as the value of wood consumption might be reported with error. Second, the estimate might be upward biased, if uptake is correlated with unobservables that are associated with less wood consumption, such as astuteness. The bias could also be in the opposite direction, if Roumdé stoves are adopted by breweries that have unobservable characteristics that are associated with lower efficiency. However, the similarity of the saving rates identified through the matching estimator on the one hand and the difference-in-difference estimator on the other hand, suggests that the bias that stems from unobserved heterogeneity is probably not too large. We have also re-estimated the matching estimator just using the sub-sample of observations used for the difference-in-difference estimator and find very similar results to those shown in Table 6 (columns 1 and 2) adding further credibility to our estimates. For example for the saving rate associated with the use of a Roumdé, we then find instead of a coefficient of -0.182 (cf. Table 6) a coefficient of -0.196 if the first set of weights is used and instead of a coefficient of -0.187 (cf. Table 6) a coefficient of -0.218 if the second set of weights is used. Yet, there could still be a problem of reverse causality: breweries with a lower consumption of firewood per litre of dolo produced are in a better position than less efficient breweries to invest in an improved stove, leading again to an overestimated saving rate. The latter is particularly relevant if credit markets fail, which in the case of dolo breweries might be often the case. In sum, given the similarity of results in the difference-in-differences and the cross-sectional estimation and since potentially remaining biases work in opposite directions, there are good reasons to believe that the above estimate is sufficiently close to the true saving rate. This is also confirmed by the fact, that the gap between the saving rate actually achieved and the potentially possible saving rate is in line with the found gap for household cooking stoves. [24] find that users of Roumdé cooking stoves realize firewood savings relative to traditional three-stones of roughly 24%, whereas the potential saving, as indicated through controlled cooking tests, stands at 40%.

A look at the included control variables (see S3 Table) reveals some further interesting insights. First and not surprisingly, the production parameters such as the quantity of malt and water used and the number of cauldrons employed matter a lot for firewood consumption. Second, whether the wood was bought from a small retailer or a sort of “wholesale dealer” has only little effect. Third, the dolotière’s education level has also no effect. Fourth, the location in Ouagadougou or Bobo-Dioulasso matters, as this does not only capture differences in the price of firewood, but also differences in the way dolo is produced. Obviously, the production parameters such as the quantity of malt and water used and the number of cauldrons employed might all be endogenous with respect to our outcome of interest. These coefficients, however, are not our main interest; we just need to make sure that none of these variables (or their omission) biases our savings rate. In contrast, not controlling for these parameters would be very problematic, as of course wood consumption first of all depends on the volume of beer prepared.

Table 7 below converts the estimated saving rate into savings per litre of dolo produced, both in monetary terms and in terms of kg of firewood. The reported mean value of firewood consumption per litre of dolo (i.e. dividing total wood expenditures by the size of the brewing) is about CFA F 24.50, 36% of that correspond to CFA F 8.82. Using an average price of firewood of CFA F 50 per kg, allows calculating the quantity of saved firewood per litre of dolo of 0.176 kg. The total savings per brewing amount to 42.3 kg of wood or CFA F 2,117. Table 7 also provides an alternative calculation where instead of traditional stoves, improved traditional stoves are used as a reference category. Assuming two brewing processes per week, these estimates suggest that the investment in a Roumdé is amortised after 6.5 weeks if a simple traditional stove is used as reference and after 14.7 weeks if an improved traditional stove is used as reference. Given that the estimated life-span of the Roumdé is much longer, buying a Roumdé seems to be a reasonable investment as long as wood has to be bought and cannot just be collected, which seems to apply for almost all dolotières we interviewed since more than 90% in our sample reported to buy their firewood. If maintenance costs are taken into account, the amortization periods extend to 7.5 and 21.2 weeks. Note that the assumed maintenance costs correspond to those reported in the survey. Optimal maintenance would require somewhat higher re-investments, but this should then also lead to a higher saving rate. Therefore we assume here actual and not optimal costs.

**Table 7:** Wood savings related to Roumdé usage in terms of value and quantity.

	Ref.: traditional stove	Ref.: improved traditional stove
Estimated saving rate	36%	16%
Mean firewood expenditure per litre of dolo	CFA F 24.50	CFA F 24.50
Saved firewood expenditures per litre of dolo	CFA F 8.82	CFA F 3.92
Price of firewood per kg	CFA F 50.00	CFA F 50.00
Saved firewood in kg per litre of dolo	0.176 kg	0.078 kg
Average size of a brewing (median)	240 liter	240 liter
Saved firewood per brewing in kg	42.336 kg	18.720 kg
Saved firewood per brewing in CFA F	CFA F 2,116.80	CFA F 936.00
Price of a Roumdé stove	CFA F 27,500	CFA F 27,500
Weeks until amortization, 2 brewings per week assumed	6.5	14.7
Weeks until amortization accounting for maintenance costs(CFA F 30,000 assumed annually)	7.5	21.2

*Source*: Own estimations, based on Brewery Survey 2012.

Three factors seem particularly important for the realized savings with a Roumdé stove. First, in a typical brewery several other persons work next to the dolotières. Even if the dolotière has some sense of how to use the improved cook stove efficiently, the other staff members do not necessarily know. Second, even if staff knows how to use a Roumdé in principle, they may not necessarily follow these rules, but rather stick to the procedures they have always applied. As explained above brewing dolo is not just a productive activity; it is an artisanry that follows first of all a tradition where the adoption of new technologies is quite uncommon. For instance, not a single brewery has been found that brews with LPG, although that would be even more energy efficient, as the temperature could be regulated over the two days in any time according to need. Third, the field visits showed that many of the improved stoves are in a very bad condition (in fact more than what the distribution of reported quality in the survey suggests). In particular the door and the inner of the combustion chamber were often damaged, due to the common practice of forcing huge trunks through the small door. These damages obviously reduce the efficiency of Roumdés quite significantly. The latter would imply, if in the estimations above we could better account for the quality of the used stoves, the estimated saving rate should be closer to the potentially possible 60%.

### Household welfare effects

Table 8 shows the absolute and relative reduction in the price of firewood for the in total nine combinations of adoption rates and alternative price elasticities. The observed average market price for one kg of wood is CFA F 50. Using the above estimated adoption rate among Dolo breweries of 44% and hence a sudden excess supply of wood by 2,204 tons per month (see S2 File how we derive this estimate) leads to a decline of the price of wood by 14% if the price elasticity is low. For a high price elasticity the absorption of the excess supply would only induce a price increase of 5%. Given that wood is used for the preparation of food and food is a basic necessity, we believe that it is plausible to assume that the price elasticity is rather below than above one. If all Dolo breweries adopted improved stoves the price decline could be as high as 11% to 32%. Again, these computations ignore supply responses in the medium and longer term and possible second-round demand effects within the group of Dolo breweries.

**Table 8:** Price changes absolute and relative (in italics) induced by wood savings among breweries.

Adoption rate (*Aggregate saving rate over all breweries*)	Assumed price elasticity of demand (ε)		
	-0.5	-1	-1.5
0.44	-7.04	-3.52	-2.35
(0.07)	*-0*.*14*	*-0*.*07*	*-0*.*05*
0.75	-12.00	-6.00	-4.00
(0.12)	*-0*.*24*	*-0*.*12*	*-0*.*08*
1.00	-16.00	-8.00	-5.33
(0.16)	*-0*.*32*	*-0*.*16*	*-0*.*11*

*Notes*: The initial average price of wood is CFA F 50.

*Source*: Own computation.

**Fig 1:**
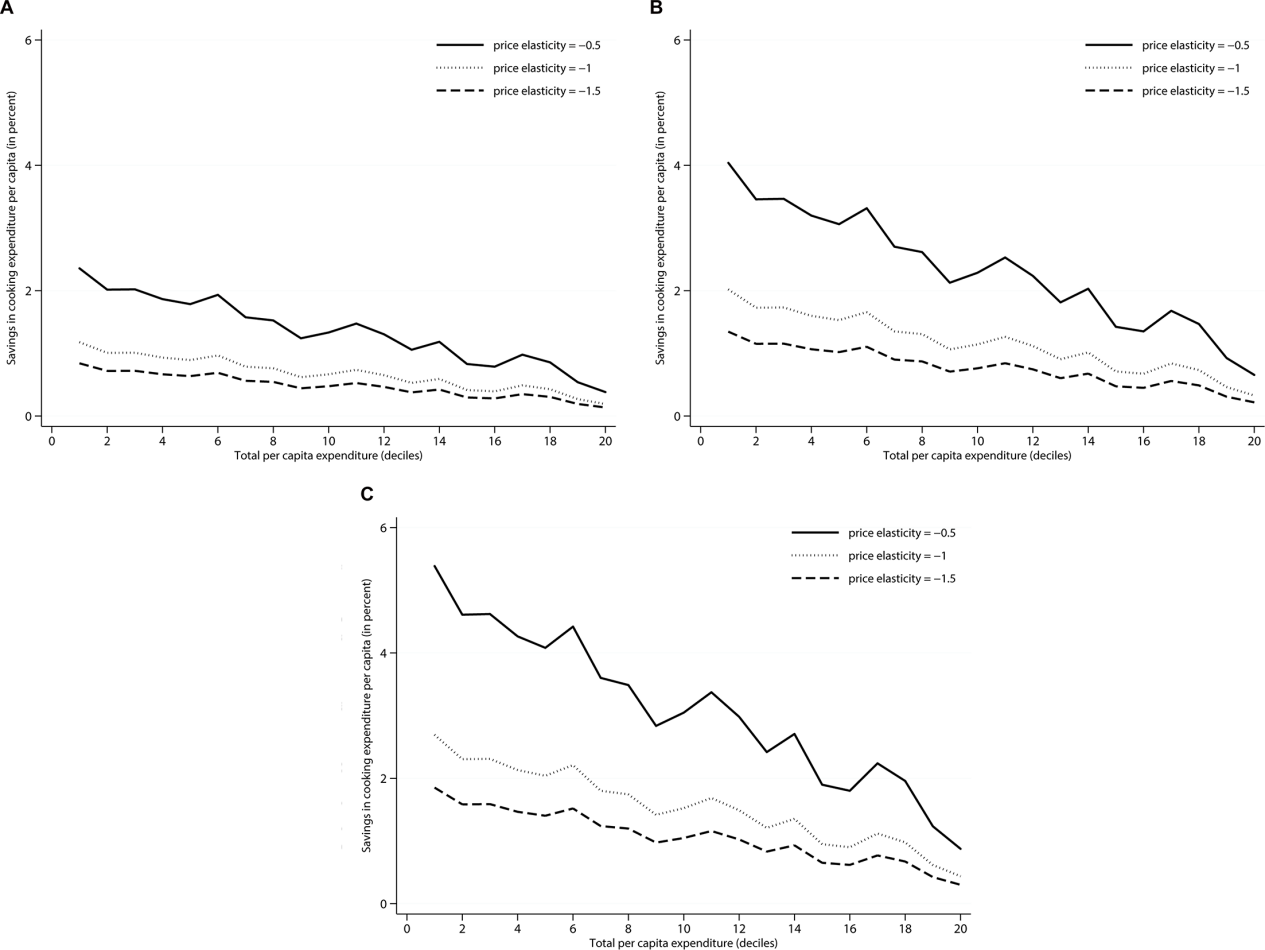
Simulated savings in cooking expenditure induced by price effect. (a) shows savings for the actual adoption rate of 44%, (b) shows savings for a hypothetical adoption rate of 75% and (c) shows savings for a hypothetical adoption rate of 100%.

To illustrate the distributional effect of the reduced price of woodfuels we draw “benefit incidence curves”, i.e. we show the relative reduction in household expenditures that is due to the reduced price. These curves are presented in Fig 1, they take into account the budget share spent on wood for each single household. Obviously, households that spend a relatively large share of their budget on woodfuels save relatively more than households that consume only little. To keep the analysis simple we apply the same saving rates for firewood and charcoal. Fig 1(A) shows the savings along the household expenditure distribution for the actual adoption rate of 44%. Households in the lowest quintiles save up to 2.5% of their total expenditures if the price elasticity is low. If it is high it is rather 1%. Savings decline with increasing expenditures as households spend lower shares of their budget on wood, partly because cooking energy increase under-proportionally with total expenditures and partly because richer households use gas or other non-wood fuels for cooking. Overall the distribution of improved stove among dolo breweries has via the market for wood a clearly pro-poor effect on consumers. If all dolo breweries would adopt an improved stove poor households could save up to 6% in their household expenditures. The traders of woodfuel obviously may incur a welfare loss, but this is unlikely to reduce the pro-poorness of the price effect.

### Environmental benefits

As outlined above, firewood usage has environmental implications on the regional level in terms of deforestation and the global level in terms of climate relevant emissions. Using the UNFCCC methodology to calculate the net CO_2_ savings induced by energy efficiency measures in thermal applications we concentrate on the reduction of CO_2_ emissions. The methodology does so far not include black carbon emissions and, instead, relies on three decisive figures: the quantity of wood saved by the intervention, the share of this savings that is non-renewable (burning renewable wood is obviously CO_2_-neutral), and the amount of CO_2_ that is emitted when burning wood. These three factors are determined in the following guided by the UNFCCC methodology.

In order to derive the quantity of wood that is saved by using the improved cooking device, UNFCCC requires taking into account an adequate baseline scenario and real-world usage behaviour. Three approaches are enumerated, the controlled cooking tests (CCT) and the water boiling tests (WBT) are laboratory tests, while the third option, the kitchen performance tests (KPT), is a field test that involves intense monitoring. It can be expected that such controlled laboratory tests or intensively monitored field tests yield higher savings rates than what can be effectively observed in the field. Therefore, we use the survey data that we analysed in the previous sections. The total wood savings we obtained for the two cities–around 26,500 tons per year–thus constitute a conservative estimation within the UNFCCC methodology.

Second, the *fraction of non-renewable biomass* (fNRB), i.e. the amount of wood that can be considered as non-renewable needs to be determined. Here, the UNFCCC methodology allows for using both project specific fNRB that are specifically determined for the forests from which the wood is extracted in the project area or, instead, using conservative country specific default values for the fNRB. The default fNRB for Burkina Faso is 90%. In other words, it is expected that only 10% of the wood extracted in the country is replaced by the natural growing process. In line with this, we assume that 90% of the firewood used in the Dolo breweries in our sample is extracted in a non-renewable way and thus, the net wood savings due to the improved brewing stoves amounts to 23,850 tons per year. A more conservative estimate would be to use a lower fNRB as suggested by [44]. If we assume the fNRB to be at 50% only, the net wood savings amount to 13,250 tons. If black carbon emissions were included in the calculation of net climate relevant benefits, discounting the firewood savings for the fNRB would not be necessary, since black carbon is soot and thus not sequestrated in the growth process of organic matter.

Third, the amount of CO_2_ that is emitted when the wood is burned needs to be determined. Since the carbon content of wood varies with the type of wood, the UNFCCC methodology uses a proxy and assumes that woodfuel users would gradually switch to fossil fuels for cooking; kerosene, liquefied petroleum gas (LPG), or hard coal. UNFCCC then approximates the achieved reduction in CO_2_ emissions by transferring the calorific value of the economized wood into this fossil fuel mix and takes the corresponding amount of CO_2_ per calorific unit. The UNFCCC default for wood fuel is 0.015 TJ/tonne. Hence, the calorific value of the saved wood equals 357.75 TJ or 198.75 TJ if we take the lower fNRB.

Since kerosene and hard coal are not used in Burkina Faso for cooking or brewing, we use the CO_2_ content of LPG as the emission factor, which is also a more conservative estimation given the higher CO_2_ intensity of kerosene and hard coal. The emission factor for LPG is 63.0 t CO_2_/TJ. Therefore, 22,538 tons of CO_2_ are saved (12,521 tons for the lower fNRB). Burkina Faso as a whole emitted 1,683,000 tons of CO_2_ in 2010 [45], so the achieved savings correspond to 1.3% of the emissions of the country and 0.75% if we assume a lower fNRB.

## Conclusion

In this paper we first evaluated the direct effects of an improved brewing stove program on firewood savings in local beer breweries in urban Burkina Faso and, then assessed the indirect effects of these savings on the price of firewood and hence on residential users’ welfare in the affected cities. Furthermore, we approximated the intervention’s effect on CO_2_-emissions that is achieved through a reduced deforestation and thus of carbon sinks. Since according to the Burkinabè Ministry of the Environment the breweries absorb around half of the firewood consumption in urban Burkina Faso, considerable second-round effects on the welfare of firewood users and the environment can be expected to the extent the intervention proofs to yield first-round effects on the firewood consumption of the breweries. We used two original data sets we collected among the dolotières, and among urban households. The energy efficiency intervention in fact leads to a reduction in firewood consumption of around 36% to 38%. Our impact assessment shows that a brewery that switches from no Roumdé stove to only Roumdé stoves, realizes savings in firewood per brewing by about 36% to 38%. Users of improved stoves save about 0.176kg of fuelwood per litre of dolo brewed. This is quite substantial. Yet, the saving rate is by roughly 40% smaller than what is technically possible (0.37/0.60). For the second-round effects we use the household data set, derive firewood demand and, based on alternative price elasticities simulate the price decrease to be expected following the sudden reduction in firewood consumption of the breweries. We find a reduction in the price of firewood of the order of 5% to 14%, depending on the price elasticity of wood demand. As for the environmental effect, we find that around 1.3% of the overall Burkinabè CO_2_-emissions are avoided due to the intervention.

The intervention therefore not only qualifies as a classical green growth policy, but also as pro-poor policy. It clearly eases adverse effects of a market failure (because social costs of firewood consumption are not fully internalized) and hence implies a Pareto-efficient improvement to the economy. In addition, the induced changes do not harm the poorest strata. If the effects on woodfuel prices we estimate are not overcompensated by long-term general equilibrium effects, the poorest strata will even gain from this intervention. [4] rightly pointed out that many green growth policies require post-intervention redistribution, because in spite of a Pareto-efficient improvement the poorest strata are frequently adversely affected by such interventions. This is not the case here.

Two major particularities of this intervention stand out as compared to many green growth and energy efficiency interventions as described in [4]: First, the targeted fuel—firewood—unlike fossil fuels is traded on regional markets and not on the world market. As a consequence, price effects materialize, which would not be the case if fossil fuel consumption is targeted. Even if substantial decreases in fossil fuel consumption are achieved, a country like Burkina Faso can hardly be expected to affect fossil fuel prices on world markets. Second, the targeted fuel is used by the vast majority of poor households in Burkina Faso on a daily basis and, hence, they potentially benefit from the second-round effects on firewood markets. In particular, urban areas firewood is almost always bought (and not collected as in rural areas) and the related expenditures constitute a considerable burden. Energy efficiency measures that target fossil fuels or electricity consumption would hardly benefit the poorest, since these fuels are barely used by the deprived strata.

Also beyond Burkina Faso, in particular the poorest of the poor living in cities spend substantial shares of their expenditures on woodfuels. Therefore, our findings suggest that green growth policies focussing on woodfuel sectors in developing countries offer a double dividend: they remove market failures with harmful consequences for both the local, regional and global environment and alleviate poverty among the poorest of the poor at the same time.

## Supporting Information

S1 File Photographs of different types of stoves. Fig A: Roumdé stove
Fig B: Traditional stove (most rudimentary)
Fig C: Traditional stove
(DOCX)

S2 File Externalities on fuel-wood consuming households. (DOCX)

S1 Table Test of balancing property of matching procedure. (DOCX)

S2 Table Analysis of drop-outs, probit model. (DOCX)

S3 Table Details of regressions shown in Table 6. (DOCX)
