# Supplementary material for: Beer, Wood, and Welfare ‒ The Impact of Improved Stove Use Among Dolo-Beer Breweries
Source: PLoS One. 2015 Aug 5;10(8):e0132603. doi: 10.1371/journal.pone.0132603 (PMC4526648; doi:10.1371/journal.pone.0132603)
Supplement: S1 File — Fig A: Roumdé stove Fig B: Traditional stove (most rudimentary) Fig C: Traditional stove (DOCX) [file pone.0132603.s001.docx]

## S1 File. Photographs of different types of stoves

**
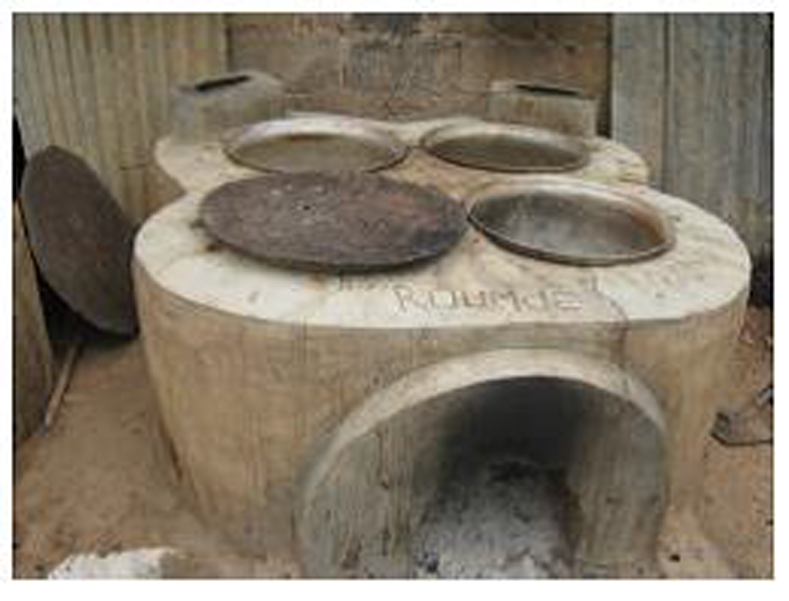
**

**Fig. A: Roumdé stove**

**
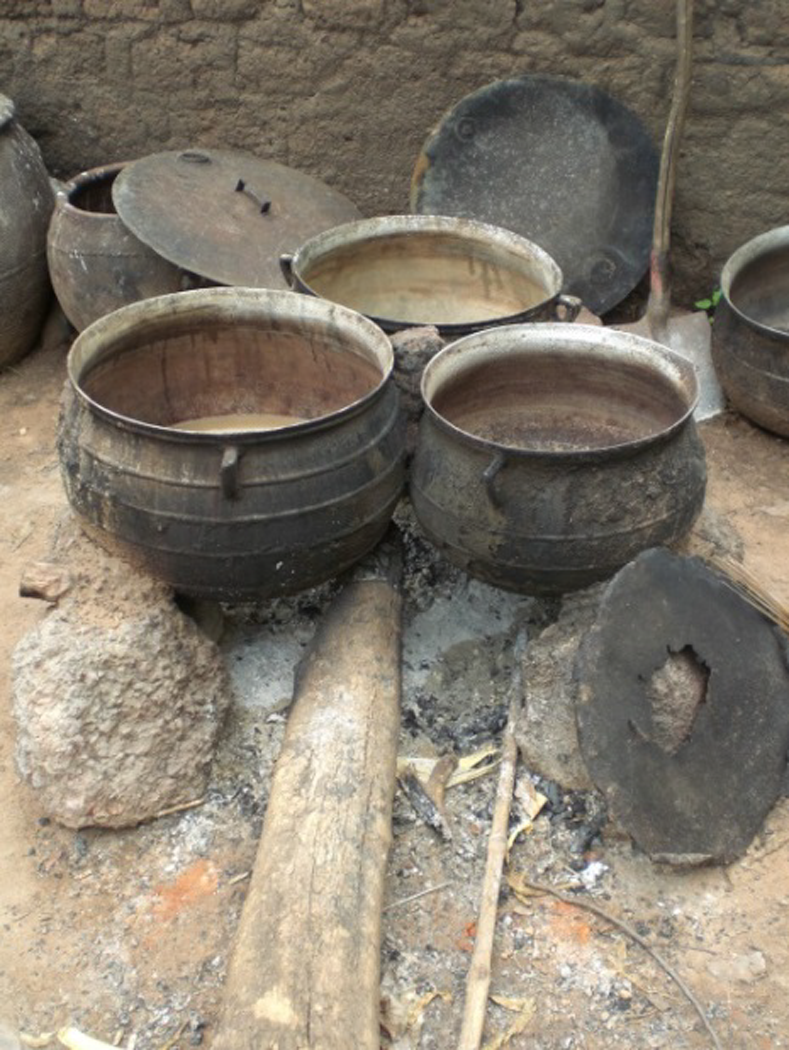
**

**Fig. B: Traditional stove (most rudimentary)**

**
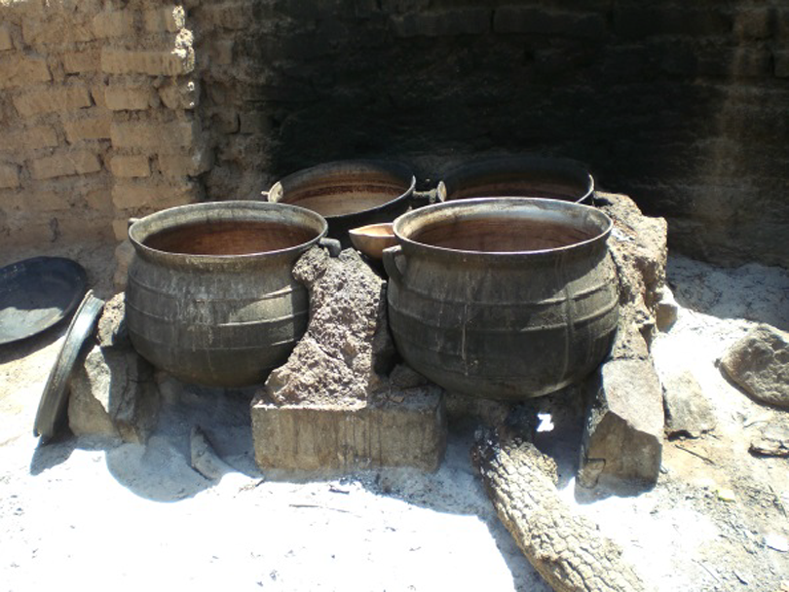
**

**Fig. C: Traditional stove**
