# Supplementary material for: Beer, Wood, and Welfare ‒ The Impact of Improved Stove Use Among Dolo-Beer Breweries
Source: PLoS One. 2015 Aug 5;10(8):e0132603. doi: 10.1371/journal.pone.0132603 (PMC4526648; doi:10.1371/journal.pone.0132603)
Supplement: S2 File — (DOCX) [file pone.0132603.s002.docx]

## S2 File. Externalities on fuel-wood consuming households

To estimate the impact of the reduced price for fuel-wood induced by the adoption of improved stoves and the resulting fuel-wood savings, we use the official estimates of the total amount of wood consumed by dolo breweries and private households (*D*) as a starting point. The Ministry of Environment assumes that Dolo breweries in Ouagadougou account for about half of the entire wood consumed in Ouagadougou. Hence, to compute the corresponding amount of wood we use the information on wood consumption in our household data. We upscale this information using the estimated population size for Ouagadougou and our survey estimate of the average household size. This gives us the entire quantity of wood consumed by households, and given the 50% assumption, also that consumed by the dolotières. To this quantity, we then apply the monetary savings induced by the decrease of firewood consumption conditional on alternative adoption rates of improved stoves among dolo breweries. We use the actually observed Roumdé adoption rate in Ouagadougou of 44% as well as two hypothetical rates: 75% and 100%. The saving rate is in line with our estimations set at 16%. This yields the change in wood consumption among dolo breweries (*∆D*). Assuming that at least in the short term the aggregate wood supply is not reduced we calculate the price change (d*p*) that is necessary that the wood saved by dolo breweries is completely absorbed by private households. The latter obviously requires an estimate of the price elasticity of demand, *ε*. As we do not have data that would allow estimating such elasticities in a reliable way, we alternatively assume a price elasticity of demand of -0.5, -1 and -1.5. An elasticity of -0.5 for instance, means that households increase their demand by 0.5% if the price decreases by 1% and so on. The induced price change is given by:

$dp=\frac{p\Delta D}{D\varepsilon}$. (S1)

We then simply estimate the welfare effect resulting from savings in wood expenditure, d*W*, by multiplying for each household in our sample the induced price change, d*p*, with the quantity of wood consumed, per household, *q*, i.e.:

$dW=qdp$*.* (S2)
