# Supplementary material for: Beer, Wood, and Welfare ‒ The Impact of Improved Stove Use Among Dolo-Beer Breweries
Source: PLoS One. 2015 Aug 5;10(8):e0132603. doi: 10.1371/journal.pone.0132603 (PMC4526648; doi:10.1371/journal.pone.0132603)
Supplement: S1 Table — (DOCX) [file pone.0132603.s003.docx]

## S1 Table. Test of balancing property of matching procedure

|  |  | Non-user of | |  |
| --- | --- | --- | --- | --- |
|  | User of | Roumdé stove | | Pre-weighing |
|  | Roumdé stove | Unweighted | weighted | difference |
| *Variable set weights I* |  |  |  |  |
| Age dolotière | 46.911 | 45.340 | 47.512 |  |
| Age dolotière (sq.) | 2313.149 | 2168.948 | 2376.088 |  |
| At least primary completed (=1) | 0.388 | 0.135 | 0.361 | *** |
| In Dolo business (years) | 18.553 | 15.151 | 17.725 | *** |
| In Dolo business (years) (sq) | 432.553 | 325.818 | 408.905 | ** |
| Ouagadougou/Centre Region | 0.854 | 0.381 | 0.849 | *** |
| Urban (=1) | 0.330 | 0.160 | 0.327 | *** |
| Ouagad. X Urban (Interaction) | 0.194 | 0.116 | 0.187 | ** |
| *Variable set weights II (those above and …)* | | | | |
| Ln quantity of Dolo per brewing (in liter) | 5.753 | 5.313 | 5.737 | *** |

*Note:* Difference: * significant at 10%. ** significant at 5%. *** significant at 1%. Values for Roumdé stove users are identical before and after weighting as a weight of 1 is assigned to these observations.

*Source:* Own estimations. based on Brewery Survey 2012.
