# Supplementary material for: Beer, Wood, and Welfare ‒ The Impact of Improved Stove Use Among Dolo-Beer Breweries
Source: PLoS One. 2015 Aug 5;10(8):e0132603. doi: 10.1371/journal.pone.0132603 (PMC4526648; doi:10.1371/journal.pone.0132603)
Supplement: S2 Table — (DOCX) [file pone.0132603.s004.docx]

## S2 Table. Analysis of drop-outs, probit model

| Dep. var.: interviewed in 2012 given that an interview took place in 2010 (=1) | Coeff.  (S.E.) | Coeff.  (S.E.) | Coeff.  (S.E.) |
| --- | --- | --- | --- |
| Age | 0.008 | 0.005 | 0.003 |
|  | (0.011) | (0.011) | (0.011) |
| Mossi (=1) | -0.287 | -0.145 | 0.053 |
|  | (0.405) | (0.419) | (0.431) |
| Bobo (=1) | -0.252 | -0.203 | -0.127 |
|  | (0.505) | (0.515) | (0.524) |
| At least primary completed (=1) | 0.584 | 0.439 | 0.379 |
|  | (0.242)** | (0.252)* | (0.259) |
| In Dolo business (years) | -0.007 | -0.006 | -0.005 |
|  | (0.012) | (0.012) | (0.013) |
| Ouaga | 0.479 | 0.32 | 0.133 |
|  | (0.461) | (0.472) | (0.487) |
| Knows, doesn't have impr. stove (in 2010) | | 0.792 | 0.767 |
|  |  | (0.221)*** | (0.224) *** |
| Has improved stove (in 2010) | 0.328 | 0.731 | 0.765 |
|  | (0.268 | (0.292)** | (0.295)*** |
| Ln of quantity dolo produced (in 2010) | 0.501 | 0.428 | 0.231 |
|  | (0.219) ** | (0.222)* | (0.240) |
| Does also retailing |  |  | 0.606 |
|  |  |  | (0.253)** |
| Constant | -3.546 | -3.405 | -2.397* |
|  | (1.182) *** | (1.196)*** | (1.285) |
| N | 192 | 192 | 192 |

*Notes:* Due to missing information in the characteristics. 25 observations had to be excluded from this regression.

*Source:* Own estimations. based on Brewery Surveys 2010 and 2012.
