# Supplementary material for: Beer, Wood, and Welfare ‒ The Impact of Improved Stove Use Among Dolo-Beer Breweries
Source: PLoS One. 2015 Aug 5;10(8):e0132603. doi: 10.1371/journal.pone.0132603 (PMC4526648; doi:10.1371/journal.pone.0132603)
Supplement: S3 Table — (DOCX) [file pone.0132603.s005.docx]

## S3 Table. Details of regressions shown in Table 6

|  | OLS-CS 2012 | OLS-CS 2012 | OLS-CS 2012 | OLS-CS 2012 | Diff-in-Diff |
| --- | --- | --- | --- | --- | --- |
|  | PS-weights I | PS-weights II | PS-weights I | PS-weights II | param. |
| Type of stove used^a)^ |  |  |  |  |  |
| Traditional/traditional improved stove | Ref. | Ref. |  |  | Ref. |
|  |  |  |  |  |  |
| Roumdé stove | -0.182 | -0.185 |  |  | -0.143 |
|  | (0.064)*** | (0.064)*** |  |  | (0.340) |
| Type of stove used by share of brewing days |  |  |  |  |  |
| Traditional stove |  |  | Ref. | Ref. |  |
|  |  |  |  |  |  |
| Improved traditional stove |  |  | -0.199 | -0.214 |  |
|  |  |  | (0.168) | (0.154) |  |
| Roumdé stove |  |  | -0.358 | -0.376 |  |
|  |  |  | (0.163)** | (0.153)** |  |
| Condition of stove by share of brewing days |  |  |  |  |  |
| Good | Ref. | Ref. | Ref. | Ref. |  |
|  |  |  |  |  |  |
| Cracks | 0.036 | 0.071 | 0.053 | 0.094 |  |
|  | (0.081) | (0.073) | (0.083) | (0.075) |  |
| Shaby | 0.104 | 0.070 | 0.080 | 0.071 |  |
|  | (0.135) | (0.106) | (0.116) | (0.102) |  |
| Ln Number of cauldrons | 0.428 | 0.314 | 0.434*** | 0.307*** |  |
|  | (0.120)*** | (0.096)*** | (0.124) | (0.095) |  |
| Ln quantity of Dolo per brewing (in liter) | 0.045 | 0.138 | 0.065 | 0.157 | 0.083 |
|  | (0.126) | (0.116) | (0.123) | (0.114) | (0.274) |
| Ln quantity of malt per brewing (in kg) | 0.491 | 0.404 | 0.458 | 0.371 | 1.112 |
|  | (0.205)** | (0.168)** | (0.197)** | (0.166)** | (0.272)*** |
| Ln quantity of water per brewing (in barrel) | 0.119 | 0.258 | 0.118 | 0.268 |  |
|  | (0.172) | (0.141)* | (0.165) | (0.136)* |  |
| Wood delivery (share of breweries) |  |  |  |  |  |
| Buys in small quantities | Ref. | Ref. | Ref. | Ref. | Ref. |
|  |  |  |  |  |  |
| By cart | -0.037 | -0.038 | -0.045 | -0.057 | -0.250 |
|  | (0.084) | (0.075) | (0.083) | (0.073) | (0.160) |
| By lorry | -0.007 | 0.022 | -0.029 | 0.007 | 0.268 |
|  | (0.101) | (0.095) | (0.112) | (0.104) | (0.249) |
| By truck | 0.155 | 0.102 | 0.157 | 0.102 | 0.343 |
|  | (0.079)* | (0.075) | (0.082)* | (0.077) | (0.281) |
| Age dolotière | -0.059 | -0.048 | -0.064 | -0.054 |  |
|  | (0.025)** | (0.025)* | (0.024)*** | (0.025)** |  |
| Age dolotière (sq.) | 0.001 | 0.001 | 0.001 | 0.001 |  |
|  | (0.000)*** | (0.000)** | (0.000)*** | (0.000)** |  |
| At least primary completed (=1) | 0.129 | 0.005 | 0.122 | 0.002 | -0.184 |
|  | (0.113) | (0.080) | (0.107) | (0.078) | (0.230) |

Table continues next page.

**Table A:** Table (*… continued*)

|  | OLS-CS 2012 | OLS-CS 2012 | OLS-CS 2012 | OLS-CS 2012 | Diff-in-Diff |
| --- | --- | --- | --- | --- | --- |
|  | PS-weights I | PS-weights II | PS-weights I | PS-weights II | param. |
| Ethnic group |  |  |  |  |  |
| Mossi (=1) | -0.166 | -0.233 | -0.196 | -0.257 | -0.415 |
|  | (0.131) | (0.117)** | (0.121) | (0.116)** | (0.221)* |
| Bobo (=1) | 0.165 | 0.159 | 0.141 | 0.170 | -0.719 |
|  | (0.117) | (0.110) | (0.121) | (0.112) | (0.313)** |
| Other (=1) | Ref. | Ref. | Ref. | Ref. | Ref. |
|  |  |  |  |  |  |
| In Dolo business (years) | 0.006 | -0.001 | 0.009 | -0.002 |  |
|  | (0.010) | (0.011) | (0.011) | (0.011) |  |
| In Dolo business (years) (sq) | -0.000 | -0.000 | -0.000 | -0.000 |  |
|  | (0.000) | (0.000) | (0.000) | (0.000) |  |
| Ouagadougou/Centre Region | 0.802 | 0.705 | 0.875 | 0.799 | -0.299 |
|  | (0.133)*** | (0.129)*** | (0.165)*** | (0.160) *** | (0.322) |
| Urban (=1) | 0.968 | 0.934 | 1.013 | 0.993 |  |
|  | (0.095)*** | (0.093)*** | (0.126)*** | (0.115)*** |  |
| Ouagad. x Urban (Interaction) | -0.730 | -0.730 | -0.754 | -0.772 |  |
|  | (0.127)*** | (0.119)*** | (0.142)*** | (0.129)*** |  |
| Time effect (2012) |  |  |  |  | -0.561 |
|  |  |  |  |  | (0.163)*** |
| Treatment group (user of Roumdé) |  |  |  |  | -0.038 |
|  |  |  |  |  | (0.275) |
| Intercept | 5.565 | 5.416 | 5.842 | 5.714 | -1.235 |
|  | (0.887)*** | (0.792)*** | (0.767)*** | (0.768)*** | (1.499) |
| R-squared | 0.766 | 0.806 | 0.768 | 0.807 | 0.655 |
| N | 236 | 236 | 236 | 236 | 66 |

*Notes:* * significant at 10%. ** significant at 5%. *** significant at 1%. Robust standard errors in parentheses.

*Source:* Own estimations. based on Brewery Surveys 2010 and 2012.
